# Meta-analysis of omega-3 polyunsaturated fatty acids on immune functions and nutritional status of patients with colorectal cancer

**DOI:** 10.3389/fnut.2022.945590

**Published:** 2022-11-21

**Authors:** Tinghui Yue, Kai Xiong, Jia Deng, Wenting Hu, Tianying Tan, Shuangshuang Li, Tao Yang, Tianbao Xiao

**Affiliations:** ^1^College of Clinical Medicine, Guizhou University of Traditional Chinese Medicine, Guiyang, China; ^2^Colorectal and Anal Surgery, First Affiliated Hospital of Guizhou University of Traditional Chinese Medicine, Guiyang, China

**Keywords:** colorectal cancer, omega-3 polyunsaturated fatty acids, humoral immunity, T cell immunity, nutritional status, meta-analysis

## Abstract

This meta-analysis assessed the clinical significance of omega-3 polyunsaturated fatty acids (PUFAs) in the management of patients with colorectal cancer (CRC) after radical resection. We comprehensively searched electronic databases, such as EMBASE, PubMed, MEDLINE and Cochrane Library, China National Knowledge Infrastructure (CNKI), China Biomedical Database (CBM), Wanfang Electronic Database, and VIP Medical Information System (VIP) from inception to 10 April 2022. Randomized controlled trials (RCTs) of omega-3 PUFAs and conventional nutrition or blank treatments were selected. The following were evaluated in the pooled analysis: immune function-related indices (IgA, IgG, IgM, CD3^+^, CD4^+^, CD8^+^, and ratio of CD4^+^/CD8^+^), nutritional status-related indices [total protein (TP), albumin (ALB), and prealbumin (PA)], and their corresponding 95% confidence intervals (CIs). Next, we conducted heterogeneity detection, sensitivity analysis, contour-enhanced funnel plot to detect possible publication bias, and meta-regression analysis. In all, 20 studies, including 1,613 patients (809 in the omega-3 PUFAs group and 804 in the control group), were selected in the final analysis. The results of the pooled analysis showed that omega-3 PUFAs significantly increased the humoral immune function indices, including IgA [standardized mean difference (SMD) = 0.54, 95% CI 0.10–0.99], IgM (SMD = 0.52, 95% CI 0.05–0.99), IgG (SMD = 0.65, 95% CI 0.47–0.84); T cell immune function indices, including CD3^+^ (SMD = 0.73, 95% CI 0.54–0.92), CD4^+^ (SMD = 0.76, 95% CI 0.53–0.98), and ratio of CD4^+^/CD8^+^ (SMD = 0.66, 95% CI 0.39–0.92). However, CD8^+^ was markedly reduced after intervention of omega-3 PUFAs (SMD = –0.28, 95% CI –0.66–0.09). In addition, pooled analysis indicated that omega-3 PUFAs markedly improved the nutritional status indicators, including TP (SMD = 0.53, 95% CI 0.17–0.88), ALB (SMD = 0.43, 95% CI 0.15–0.70), and PA (SMD = 0.46, 95% CI 0.01–0.90). The meta-regression analysis revealed that the covariates of the small sample affected the robustness and credibility of the CD4^+^ results. Conclusively, this study suggested that omega-3 PUFAs have the potential to be used as a valid immunonutritional therapy/support for treating patients with CRC postoperatively. This meta-analysis protocol was registered in PROSPERO (no. CRD42021288487).

**Systematic review registration:**

[https://www.crd.york.ac.uk/prospero/display_record.php?ID=CRD42021288487], identifier [CRD42021288487].

## Introduction

Colorectal cancer (CRC) is the most malignant tumor of the digestive system and threatens human health worldwide. The global statistical data reveal that there are approximately 1,148,515 and 732,210 new cases of colon cancer and rectal cancer, respectively, and the mortality rate of patients with CRC is approximately 9.4% (1). Furthermore, according to the American Cancer Society, CRC ranks third in the incidence of cancer among the US population. In 2021, there were 104,270 new cases of colon cancer and 45,230 new cases of rectal cancer in the United States. From 2012 to 2016, patients with CRC < 50 years increased by 2% per year while those between 50 and 64 increased by 1% per year in the United States (2). By 2035, the mortality rate from colon cancer is expected to increase by 60% and the death rate from rectal cancer is expected to increase by 71.5% (3).

The National Comprehensive Cancer Network guidelines (4, 5) and the European Society for Medical Oncology guidelines (6, 7) recommend radical surgery as a first-line treatment regimen for patients with CRC. However, the long time spent by the CRC tumor before radical resection, stress responses caused by surgical trauma, and insufficient nutritional intake may make patients susceptible to malnutrition, reduced immune function, postoperative complications, and intestinal dysfunction. Previous research revealed that the incidence of malnutrition in patients with cancer was 15–45% at the time of diagnosis, and in advanced cases, it was 80–90% (8). Additionally, the prevalence of malnutrition in patients with CRC was between 45 and 60% (9) and markedly increased with radical surgery (10). Furthermore, surgery-induced immunosuppression and immune dysfunction significantly trigger postoperative complications. Numerous studies have attributed malnutrition and immune dysfunction to postoperative complications, such as surgical site infection, anastomotic leak, intra-abdominal abscess, ureteral injury, bleeding, enteric fistula, and postoperative bowel obstruction (11–14). Thus, these complications significantly increase the hospital stay and associated medical costs as well as significantly decline the patient’s quality of life and increase cancer recurrence (15, 16).

Accumulating clinical research indicates that immunonutritional therapy/support is highly effective for enhancing the nutritional status, improving immune functions, and reducing syndromes or recrudesce in patients with postoperative CRC (17–20). Omega-3 polyunsaturated fatty acids (PUFAs) are key immunonutrients that are an essential source of energy for the intestines and thereby improve intestinal functions. Studies have shown that omega-3 PUFAs have inhibitory and lethal effects on a wide variety of tumors, such as colorectal, prostate, and breast cancers (21); In addition, long-term intake of high levels of omega-3 PUFAs can effectively reduce the incidence of CRC, breast cancer, and other malignant tumor diseases (22). Furthermore, omega-3 PUFAs improve the nutritional levels after radical resection of CRC in addition to inhibiting the inflammatory response. Omega-3 PUFAs are known for the immunostatic regulation of nutrients and improve the nutritional status of patients by accelerating the synthesis of serum albumin. Moreover, they reduce the common adverse reactions associated with enteral nutrition therapy, such as nausea, vomiting, and abdominal pain, to maintain normal gastrointestinal functions and good nutritional status, thereby improving the prognosis and accelerating the recovery of patients (23). In contrast, other studies showed that omega-3 PUFAs do not markedly improve the quality of life and postoperative complications in patients with CRC (24–26). However, there are no specific guidelines for the application of omega-3 PUFAs, in terms of time and dosage of supplementation, for patients with CRC, even the European Society for Clinical Nutrition and Metabolism (ESPEN) guidelines. The current clinical studies are heterogenous in study populations, study designs, sample quantities, and systematic approaches; hence, it is difficult to cross-examine them. Therefore, this study conducted a meta-analysis of randomized controlled trials (RCTs) in patients with CRC who received omega-3 PUFAs after radical surgery to resolve these ambiguities and assess the clinical significance of omega-3 PUFAs in these patients. Moreover, it provided substantial evidence of the effects of omega-3 PUFAs on immune functions and nutritional status in patients with postoperative CRC.

## Materials and methods

### Protocol registration

We previously registered the protocol in PROSPERO in January 2022 (number: CRD42021288487, https://www.crd.york.ac.uk/PROSPERO).

### Inclusion criteria

The type of study was RCTs clinically involving omega-3 PUFAs in patients with postoperative CRC. The inclusion criteria should be in line with the principles of “PICOS” and be qualified as follows: (1) P: the subjects of study were definitively diagnosed with CRC (including colon and rectal cancer) and underwent radical surgery. (2) I: the experimental group added omega-3 PUFAs to the control group treatment, if the two groups of subjects received general adjuvant therapy at the same time, the adjuvant therapy should be completely consistent. (3) C: the control group was treated with conventional nutrition or blank treatment (fluid supportive therapy). (4) O: the primary outcome measures included immune function-related indicators (IgA, IgG, IgM, CD3^+^, CD4^+^, CD8^+^, and the ratio of CD4^+^/CD8^+^). Secondary outcome measures included those associated with nutritional status [including total protein (TP), albumin (ALB), and prealbumin (PA)]. (5) S: patients can come from outpatient clinics or wards, and the hospital level is not limited.

### Exclusion criteria

The exclusion criteria were as follows: (1) there are repetitive publications in the literature. (2) Clinical case reports, animal experiments, review papers, letters, laboratory studies, meta-analysis, reviews, or conference papers. (3) Studies with unclear diagnostic criteria and efficacy criteria. (4) Literature with incomplete or erroneous data that cannot be combined.

### Search methodology

The PubMed, EMBASE, MEDLINE and Cochrane Library, Chinese Biomedical Database (CBM), China National Knowledge Infrastructure (CNKI), Wanfang electronic databases, and VIP medicine information system (VIP) were comprehensively searched until 10 April 2022. According to the search strategies of different databases, the search was carried out by combining titles, keywords, abstracts, subject words, and the following free words: (Colon/Rectal/colorectal/neoplasm/carcinoma/tumor) AND (Omega-3 PUFAs/Ω3-PUFAs/n-3 PUFAs/Omega 3 fatty acids/Omega-3 polyunsaturated fatty acid/Fish oil) AND (immune/immunity/IgA/IgG/ IgM/CD3^+^/CD4^+^/CD8^+^/the ratio of CD4^+^/CD8^+^) AND (nutrition/nourishment/sustenance/diet/TP/Total protein/ALB/albumin/PA/prealbumin) AND (random/randomized/clinical trial/RCTs). Furthermore, citations that may be relevant were also obtained manually, and this search strategy was not limited by the language of the publications.

### Risk of bias in literature screening, data extraction, and inclusion studies

Two researchers (Tinghui Yue and Kai Xiong) independently screened the literature according to the exclusion criteria for inclusion and used the data extraction table for data extraction. The contents of the extraction table include (1) the basic information of the included studies, including the author’s name and publication time. (2) The basic characteristics of the study subjects (male and female), such as the number of participants, age, and number of cases in each group. (3) The specific measures and timing of the intervention. (4) The outcome indicators concerned. (5) The key elements of bias risk assessment. Bias risk assessment for inclusion of RCTs: Cochrane 5.1.0 bias risk tool was used to assess the methodological quality of the implementation literature (27), and the RevMan 5.3 software was used to generate bias risk plots. The content includes random sequence generation, allocation concealment, blinding implementation, data integrity, selective publication, and other biases (small sample size, non-equilibrium baseline), which can be divided into three levels: “low risk,” “unclear,” and “high risk.” Cross-check the above results and discuss solutions if there are differences.

### Statistical analysis

Stata version 15.0 (Stata) was used for data analysis. The immune function and nutritional status-related indicators observed in this institute are continuous variables using standardized mean difference (SMD) and its 95% confidence interval (CI) as the statistical effect amount. Before determining pooled effects, heterogeneity between the included literature was assessed using the *Q*-test and the *I*^2^ test. If *I*^2^ ≤ 50% and *P* ≥ 0.1, the heterogeneity between the studies was better, and a fixed-effects model was selected for pooled analysis, and if *I*^2^ > 50%, *P* < 0.1, there is statistical heterogeneity in the results, a random-effects model was selected for pooled analysis (28, 29). The significance of pooled effects was determined using a *Z*–test, and *P* < 0.05 indicates a statistically significant difference. Sensitivity analysis was performed to verify the robustness of the combined results; make contour-enhanced funnel plot to analyze whether there was potential publication bias in the included studies.

## Results

### Results of articles screening

**FIGURE 1:**
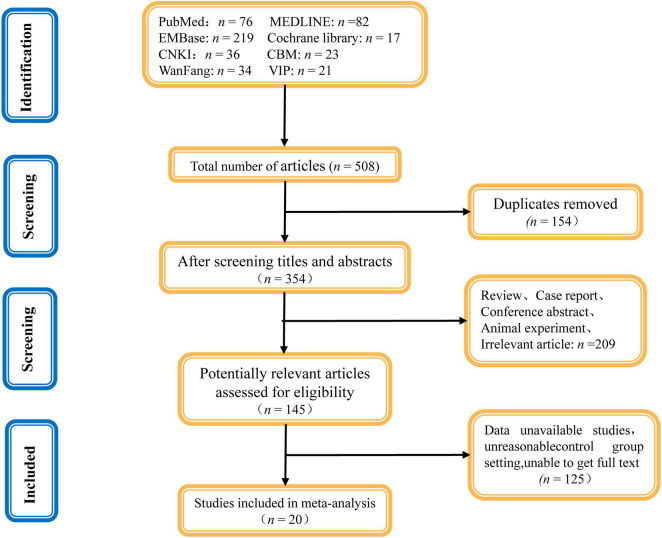
Literature screening flowchart.

In all, we acquired 508 related studies from different databases. All documents were imported into the EndnoteX9 document management software and 154 duplicate studies were excluded. After reviewing the title and abstract, 209 unrelated studies were excluded for being review articles, conference abstracts, animal experiments, or case reports. Of the remaining 145 articles, 125 articles were excluded after a full-text review based on the inclusion and exclusion criteria. Finally, 20 articles were included in the meta-analysis. Figure 1 depicts the literature screening flowchart.

### Study features

In all, 1,761 patients participated in the 20 studies (22, 24, 30–47), which were published between 2002 and 2021 years Among them, 809 were assigned to the omega-3 PUFAs group and 804 were assigned to the conventional nutrition or blank group. Four trials (30–33) administrated omega-3 PUFAs through enteral nutrition (EN) while 16 trials (22, 24, 34–47) administrated *via* parenteral nutrition (PN). Regarding the results of humoral immunity-related indicators, seven experiments (22, 24, 30, 32, 33, 43, 46) reported IgA indicator, six trials (22, 24, 30, 33, 43, 46) reported IgM indicator, and seven trials reported IgG indicator (22, 24, 30, 32, 33, 43, 46). Furthermore, concerning the results of T-cell immunity-related contents, seven trials reported CD3^+^ content (22, 24, 31, 33, 39, 42, 46), 10 trials reported CD4^+^ content (22, 24, 31, 33, 39, 42, 43, 45–47), 9 trials reported CD8^+^ content (22, 24, 33, 39, 42, 43, 45–47), and 9 trials reported the ratio of CD4^+^/CD8^+^ (22, 24, 31, 33, 39, 42, 43, 45, 47). As for the outcomes of nutritional status, 9 trials (22, 30, 31, 36–38, 40, 44, 46) reported TP, 15 trials (22, 30–34, 36–38, 40, 41, 43–46) reported the ALB indicator, and 11 trials (22, 30, 32–35, 37, 38, 40, 41, 43) reported PA indicator. The main features of the included studies material are shown in Table 1.

**TABLE 1:** The main features of the included studies material.

Study ID	Sample size (n)		Ages (year)		Dose of omega-3 PUFAs	Route of administration	Tumor types	Nationality	Type of omega 3 PUFAs administered	Outcomes
	Treatment	Control	Treatment	Control						
Braga et al. (34)	50 (M/F: 28/22)	50 (M/F: 29/21)	60.5 ± 11.5	62.2 ± 10.4	POD_1–5_: 3.3 g/L	PN	CRC (CC = NR, RC = NR)	UK	NR	➈➉
Gianotti et al. (37)	101 (M/F: 60/41)	102 (M/F: 56/46)	65.6 ± 11.5	63.4 ± 11.9	POD: 1 L/d	PN	CRC (CC = NR, RC = NR)	UK	NR	➇➈➉
Liang et al. (39)	20 (M/F: 10/10)	21 (M/F: 15/6)	59.19 ± 10.61	55.8 ± 10.1	AOD_1–7_: 1.2 g/(kg⋅d)	PN	CRC (CC = NR, RC = NR)	China	ALA, EPA, DHA	➀➁➂➃
Liu et al. (40)	20 (M/F:15/5)	20 (M/F: 14/6)	54 ± 11.3	57 ± 9.5	AOD_1–7_: 10 g/d	PN	CRC (CC = NR, RC = NR)	China	ALA, EPA, DHA	➇➈➉
Zhu et al. (47)	29 (M/F: 16/13)	28 (M/F: 17/11)	69.8 ± 10.5	70.8 ± 6.4	AOD_1–7_: 0.2 g/(kg⋅d)	PN	CRC (CC = 40, RC = 17)	China	ALA, EPA, DHA	➁➂➃
Zhuang et al. (33)	20 (M/F: NR)	20 (M/F: NR)	43–78		AOD_1–7_: NR	EN	CRC (CC = 17, RC = 23)	China	ALA, EPA, DHA	➀➁➂➃➄➅➆➇➈➉
Cai et al. (32)	20 (M/F: NR)	20 (M/F: NR)	37–76		AOD_1–7_: 3 g/d	EN	CRC (CC = 15, RC = 25)	China	NR	➄➅➈➉
Teng et al. (42)	40 (M/F: NR)	40 (M/F:NR)	42–80		AOD_1–7_: 100 mL/d	PN	CRC (CC = NR, RC = NR)	China	EPA, DHA	➀➁➂➃
Cheng et al. (36)	30 (M/F: 18/12)	30 (M/F: 19/11)	52.63 ± 6.23	53.24 ± 8.12	AOD_24weeks_: 100 mL/d	PN	CC	China	ALA, EPA, DHA	➇➈
Chen et al. (35)	49 (M/F: NR)	48 (M/F: NR)	18–70		POD_1–7_-AOD_1–3_: 2 mL/kg⋅d	PN	CC	China	NR	➉
Sun et al. (41)	48 (M/F: 30/18)	48 (M/F: 35/13)	60.1 ± 5.7	61.7 ± 6.5	AOD_1–7_: 2 ml/(Kg.d)	PN	CRC (CC = 40, RC = 56)	China	EPA, DHA	➈➉
Yespoli et al. (45)	70 (M/F: NR)	70 (M/F: NR)	59.3 ± 8.2	55.3 ± 9.1	AOD_1–7_: 1.59 g/(Kg.d)	PN	CRC (CC = NR, RC = NR)	China	NR	➁➂➃➈
Hu et al. (22)	20 (M/F: 11/9)	20 (M/F: 12/8)	62.16 ± 5.77	59.13 ± 4.43	POD_1–5_-AOD_1–7_: 100 mL/d	PN	CRC (CC = 12, RC = 28)	China	EPA, DHA	➀➁➂➃➄➅➆➇➈➉
Song et al. (31)	34 (M/F: 20/14)	34 (M/F: 19/15)	60.84 ± 6.17	60.25 ± 5.46	AOD_1–7_: 100 mL/d	EN	CC	China	ALA, EPA, DHA	➀➁➃➇➈
Wang et al. (43)	50 (M/F: 27/23)	50 (M/F: 28/22)	60.12 ± 10.14	60.24 ± 10.09	AOD_1–7_: 200 mL/d	PN	RC	China	EPA, DHA	➁➂➃➄➅➆➈➉
Jiang et al. (38)	50 (M/F: 25/25)	50 (M/F: 27/23)	61.83 ± 5.66	62.79 ± 4.87	POD_1–7_-AOD_1–7_: 2 mL/kg⋅d	PN	CRC (CC = 44, RC = 56)	China	ALA, EPA, DHA	➇➈➉
Jiang and Xie (30)	41 (M/F:25/16)	36 (M/F: 24/12)	63.05 ± 5.27	63.05 ± 5.27	POD_1–5_-AOD_1–7_: 100 mL/d	EN	CRC (CC = 39, RC = 38)	China	EPA, DHA	➄➅➆➇➈➉
Yuan et al. (46)	60 (M/F: 37/23)	60 (M/F: 33/27)	55.3 ± 7.6	54.4 ± 7.0	AOD_24weeks_: 10 mL/d	PN	CC	China	NR	➀➁➂➄➅➆➇➈
Wang and Li. (44)	27 (M/F: 15/12)	27 (M/F: 14/13)	69.69 ± 5.48		AOD_1–7_: 0.2 g/kg⋅d	PN	CRC (CC = 21, RC = 33)	China	NR	➇➈
Liu et al. (24)	30 (M/F: 18/12)	30 (M/F: 14/16)	59 ± 11	62 ± 11	AOD_1–5_: 100 mL/d	PN	RC	China	EPA, DHA	➀➁➂➃➄➅➆

NR, not report; POD, pre-operation day; AOD, after-operation day; M, male; F, female; ALA, α-linolenic acid; EPA, eicosapentaenoic acid; DHA, docosahexaenoic acid; CRC, colorectal cancer; RC, rectal cancer; CC, colon cancer; PN, parenteral nutrition; EN, enteral nutrition; ➀ CD3+; ➁ CD4+; ➂ CD8+; ➃ CD4+/CD8+; ➄ IgA; ➅ IgG; ➆ IgM; ➇ total protein (TP); ➈ albumin (ALB); ➉ prealbumin (PA).

### Study quality assessment

**FIGURE 2:**
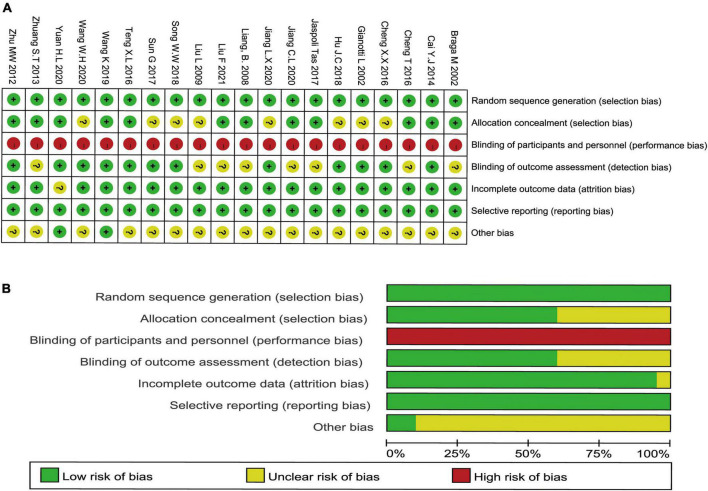
Methodological quality graph and summary of the included studies: **(A)** schematic diagram of the literature methodology quality assessment and **(B)** the ratio of literature methodology quality assessment.

The formation of the randomized sequence was recognized in all enrolled studies (Figure 2A). The allocation concealment had moderate risk in the majority of RCTs. In addition, there was no single-blinding or double-blinding in all RCTs, and none of them contained incomplete results or biased reporting. Consequently, the risk of performance bias was high, the risk of detection bias was moderate, and the implementation of concealment and blinding of some studies was unclear. Few studies did not report sample shedding, and no study selectively reported outcomes found in all trials. Figures 2A,B show the schematic diagram of the literature methodology quality assessment and the ratio of literature methodology quality assessment.

### GRADE system evaluation results

**FIGURE 3:**
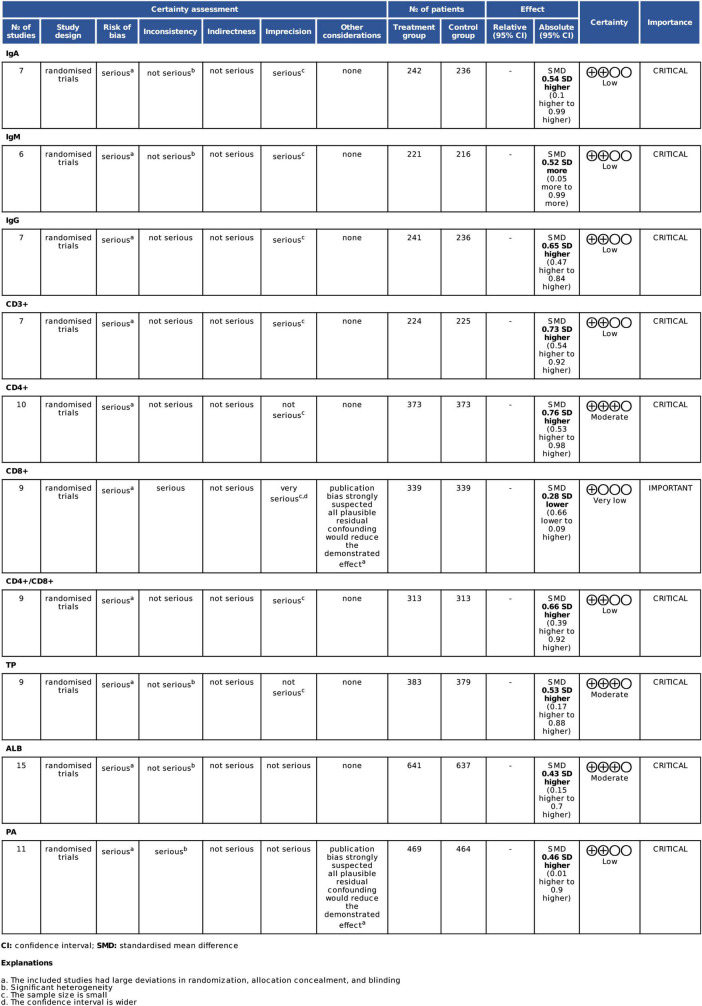
GRADE evidence profile for all outcome measures. TP, total protein; ALB, albumin; PA, prealbumin.

Using the Cochrane Collaboration Network GRADE, we assessed the quality of evidence for systematic analysis. Accordingly, we used the GRADE system to assess whether omega-3 PUFAs would enhance the immune functions and improve the nutritional status of patients with CRC after radical surgery (Figure 3). On evaluating 10 indicators, we observed that the evidence levels of IgA, IgM, IgG, CD3^+^, CD4^+^/CD8^+^, and TP were low, of CD8 + was very low, while that of CD4^+^, ALB, and PA were moderate. The decrease in evidence levels is probably because of the following reasons: (1) the included studies had large deviations in randomization, allocation concealment, and blinding; (2) significant heterogeneity (48); (3) small sample size; and (4) wide confidence interval. Downgrading to a certain degree indicates selection bias of included studies. The specific degree of heterogeneity between studies and sample size results in a downgrading of the level of evidence (Figure 3).

### Results of meta-analysis

#### Effect of omega-3 polyunsaturated fatty acids on humoral immune function in patients with postoperative colorectal cancer

**FIGURE 4:**
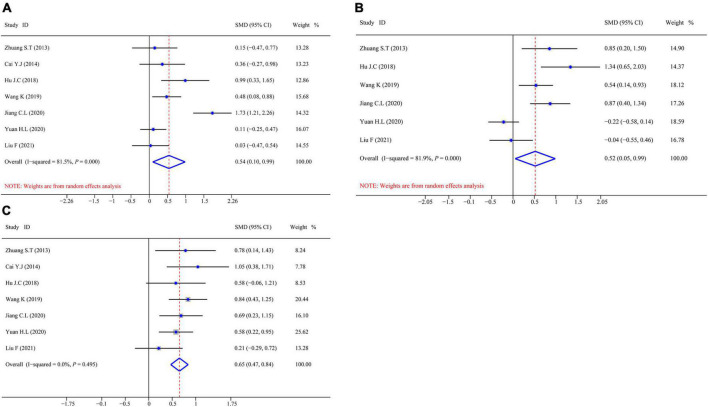
Forest plot and SMD presentation for IgA, IgM, and IgG. **(A)** Forest plot of IgA; **(B)** forest plot of IgM; and **(C)** forest plot of IgG. In all aggregated analyses, IgA and IgM used random-effects model, and IgG used fixed-effect model.

Figure 4 shows the aggregated analysis and SMD presentation of the humoral immunity-related indicators, namely, IgA, IgM, and IgG. Heterogeneity was examined before performing a pooled analysis of these indicators. The results showed a remarkable heterogeneity for IgA (*I*^2^ test = 81.5% and *Q*-test *P* = 0.000) and IgM (*I*^2^ test = 81.9% and *Q*-test *P* = 0.000, Figure 4B); thus, we performed an aggregated analysis using the random-effects model. However, there was no remarkable heterogeneity for IgG (*I*^2^ test = 0.0% and *Q*-test *P* = 0.000, Figure 4C); therefore, we performed an aggregated analysis using the fixed-effects model. We observed that the omega-3 PUFAs group had significantly higher IgA content (*Z* = 5.042, *P* = 0.000; SMD = 0.54, 95% CI 0.10–0.99; Figure 4A), IgM content (*Z* = 3.887, *P* = 0.000; SMD = 0.52, 95% CI 0.05–0.99; Figure 4B), and IgG content (*Z* = 6.930, *P* = 0.495; SMD = 0.65, 95% CI 0.47–0.84; Figure 4C) compared with the matching group. These results suggested that omega-3 PUFAs improve the humoral immune functions in patients with postoperative CRC.

#### Effect of omega-3 polyunsaturated fatty acids on T cell immune function in patients with postoperative colorectal cancer

**FIGURE 5:**
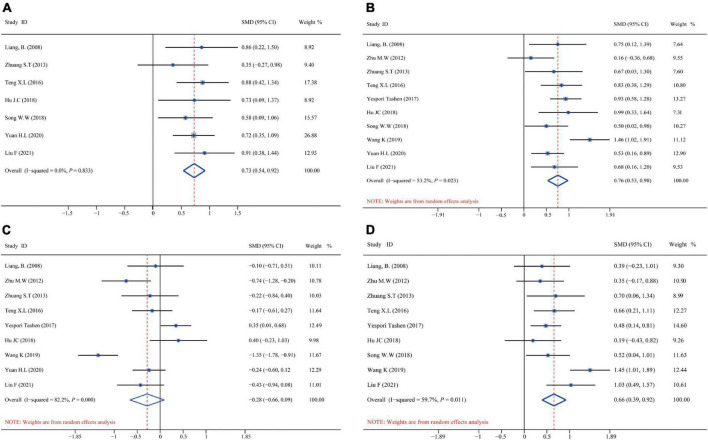
Forest plot and SMD presentation for CD3^+^, CD4^+^, CD8^+^, and CD4^+^/CD8^+^. **(A)** Forest plot of CD3^+^; **(B)** forest plot of CD4^+^; **(C)** forest plot of CD8^+^; and **(D)** forest plot of CD4^+^/CD8^+^. In all aggregated analyses, CD3^+^ used fixed-effect model, and CD4^+^, CD8^+^, CD4^+^/CD8^+^ used random-effects model.

Figure 5 shows the aggregated analysis and SMD presentation of the T-cell immunity-related indicators, namely, CD3^+^, CD4^+^, CD8^+^, and CD4^+^/CD8^+^. Routine heterogeneity tests were performed for each index before aggregated analysis. The results showed no remarkable heterogeneity for CD3^+^ (*I*^2^ test = 0.0% and *Q*-test *P* = 0.000, Figure 5A); thus, we performed an aggregated analysis using the fixed-effects model. However, there was mild heterogeneity for CD4^+^ (*I*^2^ test = 53.2% and *Q*-test *P* = 0.000, Figure 5B) and ratio of CD4^+^/CD8^+^ (*I*^2^ test = 59.7% and *Q*-test *P* = 0.000, Figure 5D), while remarkable heterogeneity for CD8^+^ (*I*^2^ test = 82.2% and *Q-*test *P* = 0.001, Figure 5C). Hence, we performed an aggregated analysis using the random-effects model. We observed that the omega-3 PUFAs group had significantly higher CD3^+^ index (*Z* = 7.465, *P* = 0.833; SMD = 0.73, 95% CI 0.54–0.92; Figure 5A), CD4^+^ index (*Z* = 10.014, *P* = 0.023; SMD = 0.76, 95% CI 0.53–0.98; Figure 5B), and ratio of CD4^+^/CD8^+^ (*Z* = 8.033, *P* = 0.011; SMD = 0.66, 95% CI 0.39–0.92; Figure 5D) compared with the matching group. In contrast, the CD8^+^ index was significantly decreased (*Z* = −3.253, *P* = 0.000; SMD = –0.28, 95% CI: –0.66 to 0.09; Figure 5C) in the omega-3 PUFAs cohort compared with the matching group.

#### Effect of omega-3 polyunsaturated fatty acids on nutritional status in patients with postoperative colorectal cancer

**FIGURE 6:**
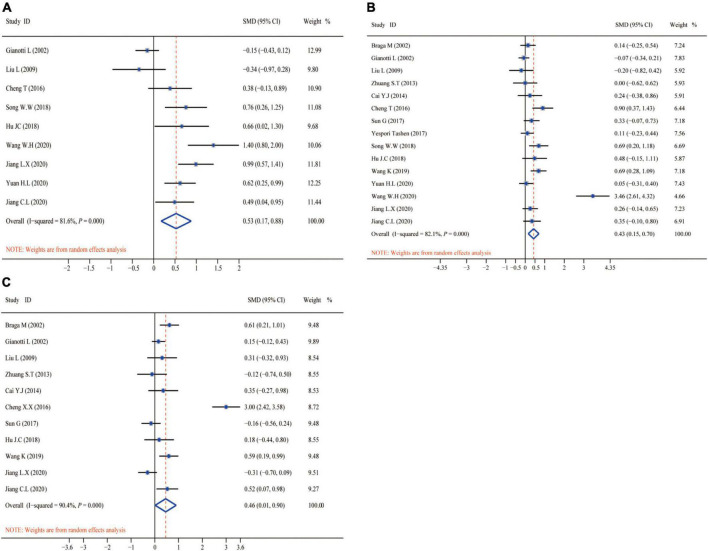
Forest plot and SMD presentation for TP, ALB, and PA. **(A)** Forest plot of TP; **(B)** forest plot of ALB; and **(C)** forest plot of PA. All aggregated analyses were used random-effect model. TP, total protein; ALB, albumin; PA, prealbumin.

Figure 6 shows the aggregated analysis and SMD presentation of the nutritional status-related indicators, namely, TP, ALB, and PA. Routine heterogeneity tests were performed for each indicator before aggregated analysis. The results showed remarkable heterogeneity for TP (*I*^2^ test = 81.6% and *Q*-test *P* = 0.000, Figure 6A), ALB (*I*^2^ test = 82.1% and *Q*-test *P* = 0.000, Figure 6B), and PA (*I*^2^ test = 90.4% and *Q*-test *P* = 0.000, Figure 6C). Accordingly, we performed aggregated analysis using the random-effects model. We observed that the omega-3 PUFAs group had significantly higher TP index (*Z* = 5.712, *P* = 0.000; SMD = 0.53, 95% CI 0.17–0.88; Figure 6A), ALB index (*Z* = 5.081, *P* = 0.000; SMD = 0.43, 95% CI 0.15–0.70; Figure 6B), and PA index (*Z* = 5.151, *P* = 0.000; SMD = 0.46, 95% CI 0.01–0.90; Figure 6C) compared with the matching group in the aggregated analysis.

#### Sensitivity analysis for the robustness of the pooled analysis

**FIGURE 7:**
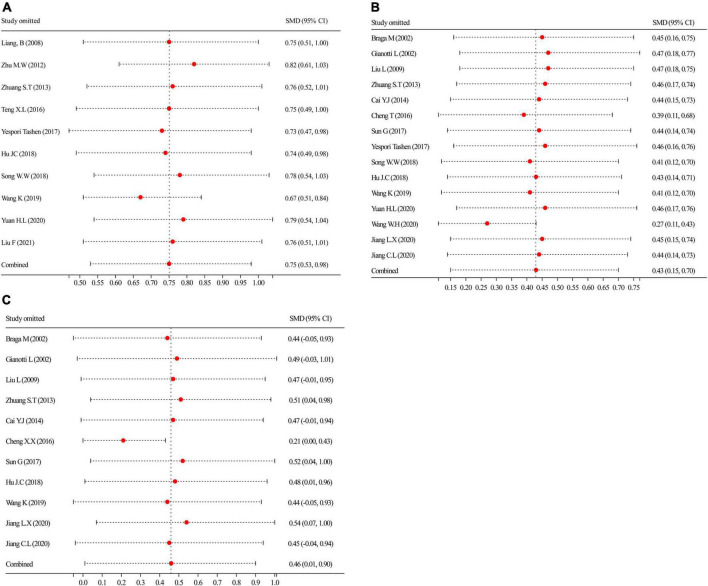
By leaving a procedure, each time was performed to carry out sensitivity analyses. **(A)** Sensitivity analysis of CD4^+^; **(B)** sensitivity analysis of ALB; and **(C)** sensitivity analysis of PA. ALB, albumin; PA, prealbumin.

Figure 7 shows the sensitivity analysis for the robustness of the pooled findings for CD4^+^, ALB, and PA by excluding a study each time and considering significant heterogeneity in the included studies having a sample size ≥ 10. Sensitivity analysis of the CD4^+^ results (Figure 7A) indicated that excluding any study did not obviously explain the heterogeneity, suggesting that the CD4^+^ aggregated outcomes were moderately robust. In contrast, the sensitivity analyses of ALB (Figure 7B) and PA (Figure 7C) showed that missing none of the studies noticeably affected the robustness of the aggregated analysis. Based on the results of the sensitivity analyses, the aggregated outcomes have a degree of robustness.

#### Contour-enhanced funnel plot to detect possible publication bias

**FIGURE 8:**
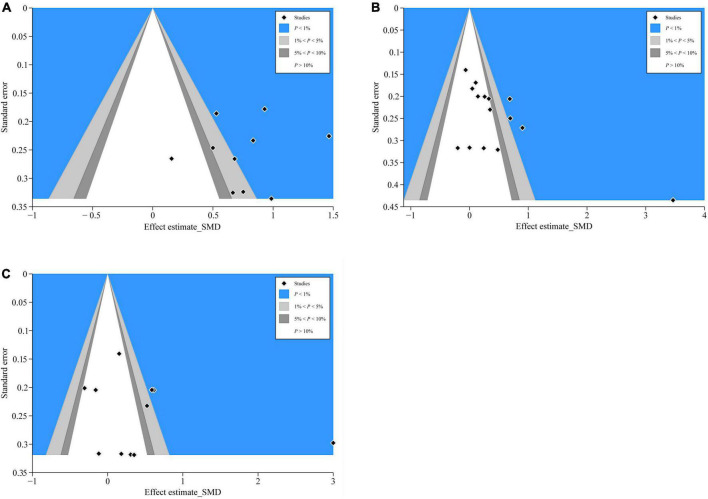
Contour-enhanced funnel plots of CD4^+^, ALB, and PA. **(A)** CD4^+^’s contour-enhanced funnel plot; **(B)** ALB’s contour-enhanced funnel plot; and **(C)** PA’s contour-enhanced funnel plot.

We distinguished the detailed causes of bias using the contour-enhanced funnel plot with the statistical significance level (*P* < 0.01, *P* < 0.05, *P* ≤ 0.1, or *P* > 0.1) and incorporated standard milestones into the funnel charts. The results of CD4^+^ (Figure 8A), ALB (Figure 8B), and PA (Figure 8C) showed that most of the missing studies occurred in higher statistically significant areas (*P* < 0.01), which suggested that the origin of asymmetry was probably due to undiscovered elements and not publication bias. Further, to explain the undetected bias, we traced back the primordial studies, speculating the small sample size, blinding missing, and intention-to-treat analysis of many studies; such factors would potentially affect our conclusions.

#### Meta-regression analysis

**FIGURE 9:**
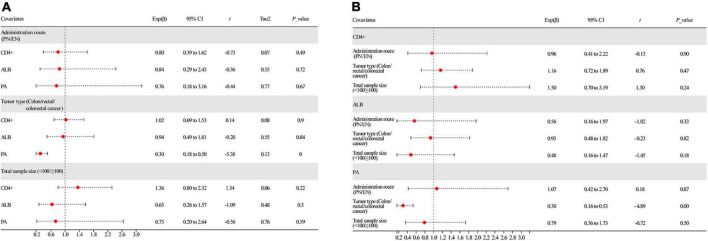
Results of meta-regression analysis. **(A)** Univariate analysis of all covariates and **(B)** multivariate analysis of all covariates. ALB, albumin; PA, prealbumin.

We performed meta-regression to assess the effect of potential confounding factors and their sources of heterogeneity on the aggregated effect estimates. These covariates are speculated to be latent elements influencing heterogeneity of the combined outcomes: (1) route of administration of omega-3PUFAs (PN or EN); (2) tumor type (colon/rectal/colorectal cancer); and (3) total sample size (<100 or ≥100). According to univariate analysis, the administration route of omega-3 PUFAs and total sample size (Figure 9A and Table 2) had no remarkable effect on CD4^+^ and ALB results (*P* > 0.05). Conversely, tumor type significantly affected the combined effect of PA (*P* = 0.00, Figure 9A and Table 2). Further, multivariate analysis was performed to assess the effect of these covariates on the combined effects. We identified that the three covariates neither affected the combined effects of CD4^+^ and ALB nor the heterogeneity stem from this model (*P* > 0.05, Figure 9B and Table 2). In contrast, multivariate analysis demonstrated that the endpoint of PA was affected by the tumor type (*P* = 0.00, Figure 9B and Table 2), indicating that the heterogeneity may be due to this covariate.

**TABLE 2:** Results of meta-regression analysis.

Covariates	Univariate analysis				Multivariate analysis		
	Exponentiated coefficient	95% CI	*P*	Tau^2^	Exponentiated coefficient	95% CI	*P*
**Administration route (PN/EN)**							
CD4^+^ (10 studies)	0.80	0.39–1.62	0.49	0.07	0.96	0.41 to 2.22	0.90
ALB (15 studies)	0.84	0.29–2.43	0.72	0.55	0.56	0.16 to 1.97	0.33
PA (11 studies)	0.76	0.18–3.16	0.67	0.77	1.07	0.42 to 2.70	0.87
**Tumor type (Colon/rectal/colorectal cancer)**							
CD4^+^ (10 studies)	1.02	0.69–1.53	0.90	0.08	1.16	0.72 to 1.89	0.47
ALB (15 studies)	0.94	0.49–1.81	0.84	0.55	0.93	0.48 to 1.82	0.82
PA (11 studies)	0.30	0.18–0.50	**0.00**	0.13	0.30	0.16 to 0.53	**0.00**
**Total sample size (<100/ ≥100)**							
CD4^+^ (10 studies)	1.36	0.80–2.32	0.22	0.06	1.50	0.70 to 3.19	0.24
ALB (15 studies)	0.63	0.26–1.57	0.30	0.48	0.48	0.16 to 1.47	0.18
PA (11 studies)	0.73	0.20–2.64	0.59	0.76	0.79	0.36 to 1.73	0.50

NA, not applicable; ALB, albumin; PA, prealbumin. Significant results are in bold and underlined presentation.

## Discussion

This study revealed that omega-3 PUFAs could significantly improve the immune functions, including humoral immune function and T-cell immune function, and nutritional status of patients suffering from CRC. We determined the levels of humoral immunity-related indicators (IgA, IgM, and IgG), T-cell immunity-related indicators (CD3^+^, CD4^+^, CD8^+^, and CD4^+^/CD8^+^), and nutritional status-related indicators (TP, ALB, and PA) at baseline and after administering omega-3 PUFAs to the treatment group. The integrated analysis revealed that IgA (SMD = 0.54, 95% CI 0.10–0.99), IgM (SMD = 0.52, 95% CI 0.05–0.99), and IgG (SMD = 0.65, 95% CI 0.47–0.84) were significantly increased in the omega-3 PUFAs group compared with the control group. These findings demonstrate that omega-3 PUFAs effectively improve the humoral immune function of patients with CRC after surgery. In addition, the aggregated analysis revealed that CD3^+^ (SMD = 0.73, 95% CI 0.54–0.92), CD4^+^ (SMD = 0.76, 95% CI 0.53–0.98), and the ratio of CD4^+^/CD8^+^ (SMD = 0.66, 95% CI 0.39–0.92) were significantly higher in the omega-3 PUFAs group (*Z* = 7.465, *P* = 0.833; SMD = 0.73, 95% CI 0.54–0.92; Figure 5A) compared with the matching group. Contrarily, CD8^+^ significantly decreased (*Z* = –3.253, *P* = 0.000; SMD = –0.28, 95% CI –0.66 to 0.09; Figure 5C) in the omega-3 PUFAs group compared with the control group. These outcomes insinuate that omega-3 PUFAs effectively improve the T-cell immune functions of patients with CRC after surgery. Moreover, the results of comprehensive analysis showed that TP (SMD = 0.53, 95% CI 0.17–0.88), ALB (SMD = 0.43, 95% CI 0.15–0.70), and PA (SMD = 0.46, 95% CI 0.01–0.90) were significantly higher in the omega-3 PUFAs cohort compared with the matching cohort. These outcomes indicate that omega-3 PUFAs effectively enhanced the nutritional status of patients with CRC after surgery. The above supporting evidence indicates that omega-3 PUFAs are conducive to improving the immune functions and nutritional status of patients. Thus, omega-3 PUFAs are an effective immunonutritional therapy/support for treating patients with CRC after radical surgery.

**FIGURE 10:**
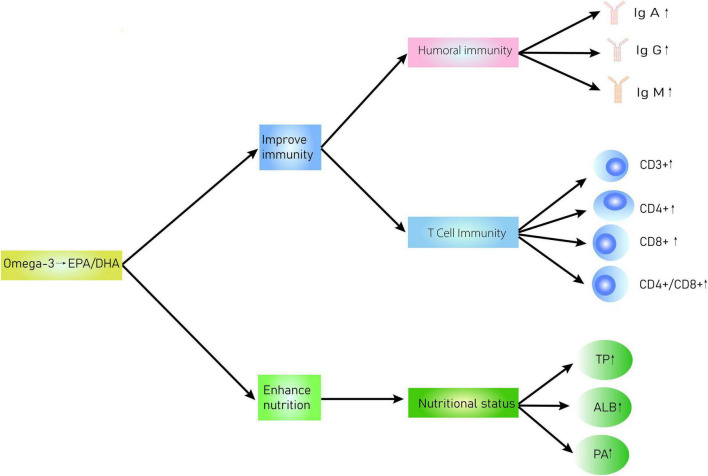
Interaction diagram of omega-3 PUFAs regulation of immune functions and nutritional status in patients with CRC.

The evaluation and application of immunonutritional therapy/support have largely been overlooked (49). So far, the ESPEN has recommended general immunonutrition support for malnourished patients with cancer (50), which coincides with the plan of Enhanced Recovery After Surgery (ERAS) (51). α-Linolenic acid (ALA), eicosapentaenoic acid (EPA), and docosahexaenoic acid (DHA) are the main components of omega-3 PUFAs (52). EPA and DHA are the main metabolites (53) and affect the structure and function of the cell membrane by competitively inhibiting the synthesis of arachidonic acid on cell membranes. Additionally, they alter the cell membrane surface receptors and regulate signal transduction, thereby modulating the inflammatory balance, inhibiting lipid peroxidation, regulating immune function, and even achieving auxiliary antitumor effects (54–57). Presently, omega-3 PUFAs are believed to play several crucial roles in the human body: (1) they inhibit arachidonic acid metabolism and thereby reduce the release of pro-inflammatory substances and thus the inflammatory response (58). They also inhibit inflammatory response by acting on cytokines related to enzymes or genes associated with the inflammatory response for decreasing the production of pro-inflammatory cytokines (59). They improve the nutritional level of patients, thereby improving the cachexia caused by cancer and the quality of life of patients (23). (2) Omega-3 PUFAs affect the integrity of specific cell membrane structures and thereon affect the normal movement of cells, receptor formation, binding of receptors and ligands, signal transduction function of cell membranes, and ultimately disrupt the production and release of cytokines, such as inhibiting the degradation of NF-κB and COX-2 and release of inflammatory factors (60). (3) Deficiency of omega-3 PUFAs can induce the expression of prostaglandins, leukotrienes, and thromboxane A2, resulting in a severe stress response that leads to immunosuppression, platelet aggregation, and excessive inflammatory response (61). Figure 10 shows the interaction of omega-3 PUFAs in regulating immune functions and nutritional status. Interestingly, a clinical trial reported that omega-3 PUFAs and glutamine can improve the immune function of patients, including CD4^+^, CD8^+^, complement C3, IgG, and IgA, and reduce inflammatory indicators (62). Accordingly, we believe that a lack of omega-3 PUFAs may impair the immune function and nutritional status of patients with postoperative CRC. However, supplementation with omega-3 PUFAs has the potential to improve the immune functions and nutritional status of patients with postoperative CRC. Nonetheless, previous studies indicate that long-term supplementation with omega-3 PUFAs may lead to gastrointestinal disturbances, bleeding, and other adverse reactions. Thus, the justification and safety of long-term high doses of omega-3 PUFA supplementation are yet to be established. Nevertheless, the prospect of omega-3 PUFA therapy is promising. To strengthen recommendations of omega-3 PUFAs therapy for patients with CRC, further studies that focus on large-scale EN or PN omega-3 PUFAs administration and its long-term use are necessary.

This study mainly focused on the clinical benefits of omega-3 PUFAs in patients with CRC after radical surgery. However, the latent boundedness of this comprehensive analysis should be highlighted. Since the 20 studies included in this meta-analysis were neither single nor double-blind, the detection bias risk must be addressed. Meanwhile, the undiscovered bias predicted by the contour-enhanced funnel plot suggested that studies with small sample sizes and a lack of intention-to-treat analysis may explain the underlying bias. Such elements may produce a latent influence on the result. Meta-regression analysis identified the sample size of the primordial research as a latent covariate, which led to prominent heterogeneity and reduced the effectiveness of the outcomes of the aggregated analysis. Although we tried to maintain the homogeneity of included studies through strict inclusion and exclusion criteria, heterogeneity was inevitable among all the studies included in the meta-analysis. The protocol of “PICOS” was different for all included studies, which lead to clinical heterogeneity. Additionally, the original studies were conducted using different RCT methods, with large or small sample sizes, etc., which lead to methodological heterogeneity. Therefore, we attributed the significant heterogeneity between included studies to the clinical and methodological heterogeneities. Taken together, this meta-analysis of 1,613 patients from 20 RCTs provided key proof that replenishment of omega-3 PUFAs was significantly effective in improving the immune functions and nutritional status of patients with postoperative CRC. However, methodological boundedness should be noted when adopting the outcomes of this research. It is widely believed that CRC management in the preoperative or postoperative phases is in dire need of the involvement of multidisciplinary teams and long-term medication. Accordingly, there is an urgent need to increase RCTs with multidimensional efficacy and large-scale nutritional status assessments to assess the risk–benefit profile of omega-3 PUFAs in postoperative CRC management.

## Data availability statement

The original contributions presented in this study are included in the article/supplementary material, further inquiries can be directed to the corresponding authors.

## Author contributions

THY performed the search and drafted the manuscript. THY and KX performed the data extraction and analyzed the data. TY and TX designed the study, drafted the manuscript preparation, and revision. JD, WH, TT, and SL were equally involved and contributed to the study conduction. All authors contributed to the article and approved the submitted version.
